# Miscellaneous Paragraphs

**Published:** 1893-12

**Authors:** 


### MISCELLANEOUS PARAGRAPHS.

### CURE FOR ROUND SHOULDERS.

A woman physician has recommended the following movements for the cure of all except very "severe cases" of round shoulders, when braces are also sometimes a necessity: "1. Raise arms before your shoulder high; extend arms sideways; throw head back; straighten head; move arms forward; lower arms; repeat ten times. 2. Stand erect; raise arms before you; rise on tiptoes, then throw arms as far backward as possible; sink again on heels, and drop arms to side; repeat ten times. 3. Raise arms with elbow bent, shoulder high, bringing palms together in front of face; then, with elbows still bent, swing both arms vigorously backward as far as possible even with the shoulders, palms looking forward. This should be repeated several times, but as the position is somewhat fatiguing, rest or change of exercise may be made between the movements." Another simple movement designed to bring about a correct position of the shoulder blades consists of holding a cane or wand in both hands, throwing the head back, and carrying the stick from "above the head back and down the hips."

As the clothing, if too tight or unyielding about or over the shoulders, may help to produce round shoulders, both the under and outside waist should be comfortable, and bands over the shoulders of garments made of elastic.

### POSITION DURING SLEEP.

There are several theories of the proper position in sleep. The one most commonly favored is that one should sleep on the right side, as digestion goes on in this position most favorably. Other anthorities say that one should always lie on the back, but there are excellent reasons why this is not wise. The weight of the stomach and its contents rests upon the spine, which often affects the nerves. Some severe cases of insomnia have been cured by the habit of sleeping on the face. This is easy to do and is the most comfortable position if one dispenses with the pillow. One young man who had exhasted all the skill of the doctors fell into the habit of lying on his face, with his right arm under his head, which was turned slightly to one side. By this change natural rest soon came to him, and he entirely recovered.

### SENDING FLOWERS BY MAIL.

There is no reason why fresh flowers should not be sent by mail much oftener than they are, if only one would go to some trouble to arrange them properly for safe carriage. They should, in the first place, be put in a wooden box such as is made expressly for that purpose and can easily be procured. But if one should chance to be where he cannot get one, a cigar box can be used. The box should then be lined with cotton batting, and on this should be placed oiled tissue paper, the object being to keep the flowers as nearly air-tight as possible. After standing in water for at least an hour or two, the flowers should be packed very closely into the box, sprinkled slightly, covered with the oiled paper and cotton batting, and the box then sealed. If these directions be followed they can be sent any reasonable distance without losing their freshness and beauty.

### SIMPLE DENTIFRICE.

Dissolve two ounces of borax in three pints of boiling water, and before it is cold add one teaspoonful of the spirits of camphor and bottle for use. A tablespoonful of this mixture, mixed with an equal quantity of tepid water and applied daily with a soft brush, preserves and beautifies the teeth, extirpates all tartarous adhesion, arrests decay, induces healthy action of the gums, and makes the teeth pearly white. The dark colored substance which collects on neglected teeth cannot be removed with a brush and water. Pulverized charcoal will take it off, but this scratches the enamel and leads to decay of the tooth. A better substance is pumice stone in powder. Dip a pine stick into it and scour the teeth. After this treatment the daily use of the tooth brush and tepid water will be sufficient.

### ABOUT SLEEPING.

Sunlight is good for everything but feathers.

The best number of persons to each bed is—one.

Away with heavy hangings, either above or below the bed.

Beware of a dusty, musty carpet; better sweetness and a bare floor.

Do not fail to provide some means of ventilation during the night.

Keep the head cool while sleeping, but not by a draught of cold air falling upon it.

If a folding bed must be used, contrive some way to keep it aired and wholesome.

Let the pillow be high enough to bring the head in a natural position—no more nor less.

Thoroughly air the sleeping-room every day; air the beds and bedding as often as possible.

A dark, out-of-the-way, unwholesome corner is no more fitted for a sleeping room than for a parlor.

A feather bed which has done service for a generation or two is hardly a desirable thing upon which to sleep.

### AT THE END OF THE DAY.

When the day has been long and hard; when a sharp pain begins to make itself felt in the busy woman's forehead and a dull ache in the back of her neck; and when she is sure that the only answer to the question as to whether life is worth living is a negative, then there is only one thing for her to do. And that one thing is not to read a cheerful or even a pious book, or to force herself into a pleasant mood. It is this:

First, she must get out of her tight clothes and bunch her hair on the top of her head. Then she must bathe her face for five minutes in the hottest water she can bear. Then her neck must be treated to the same process. She will look like a lobster or a beet, but she should refrain from looking at herself. After that she should lie down flat on her back and give herself up to the single thought of how heavy she is and how the couch supports her.

If she does not fall asleep, as she probably will, she should rise at the end of half an hour of this intellectual enjoyment. She will feel ten years younger. There will be no pain any where, and she will have a cheerful conviction that life is very much worth living. Moreover, the lobster hue will have faded to rose, and she will look as well as she feels.

Nature furnishes without cost the four most efficient disinfectants—sunshine, fresh air, water, and dry earth. With care any house properly constructed may in this clime be kept perfectly clean by the daily use of these powerful agents, which cost not a cent.

Do not worry. Eat three square meals a day. Say your prayers.—*Abraham Lincoln.*

